# Diffusive to Barrier-Limited Transition in the Aqueous Ion Transport through Nanoporous 2D Materials

**DOI:** 10.1021/acs.jpcb.5c00921

**Published:** 2025-05-06

**Authors:** Yechan Noh, Alex Smolyanitsky

**Affiliations:** † Department of Physics, University of Colorado Boulder, Boulder, Colorado 80305, United States; ‡ Applied Chemicals and Materials Division, National Institute of Standards and Technology, Boulder, Colorado 80305, United States; ¶ Department of Materials Science and Engineering, University of California, Berkeley, Berkeley, California 94720, United States

## Abstract

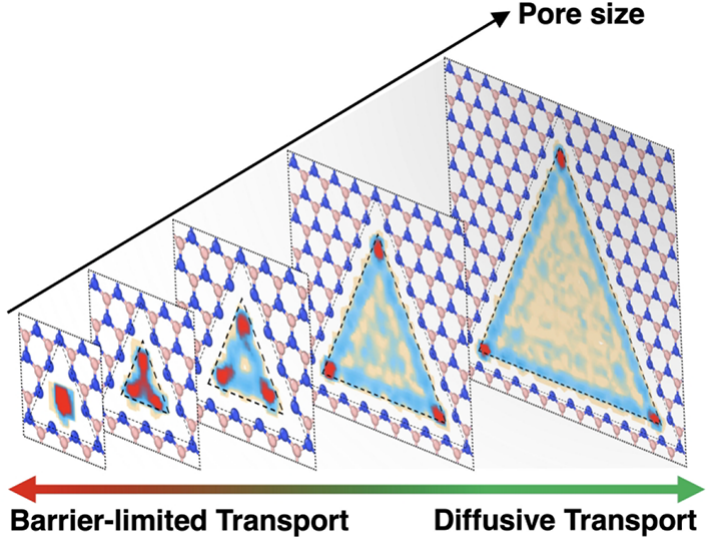

The interplay of interactions between aqueous ions and the confinement of subnanoscale pores in solid 2D membranes causes a range of barrier-limited phenomena, including selective ion trapping and permeation, mechanosensitive transport, and memristive effects. A clear understanding of the transition from diffusive to barrier-limited transport regime is lacking, however. Moreover, the limits of applicability for the analytical formalism widely used to relate measured transport data to the effective pore size are unclear. Here, with the goal of identifying the transition between regimes and determining the pore sizes below which the diffusive formalism fails, we present a computational study of water-dissociated alkali salt transport through 2D membranes featuring pores of various sizes. Triangular nitrogen-terminated multivacancies in hexagonal boron nitride are used as a simple yet illustrative example of uncharged locally dipolar pores with various degrees of cation selectivity. We find that *cation–cation* selectivity and high mechanosensitivity are the clearest indicators of the barrier-limited regime onset. We also show that for triangular pore geometries, the diffusion-based analytical formalism is expected to fail when the side of the triangle is below order ≈2 nm. For circular geometries, similar failure is expected for pore diameters below ≈1.2 nm. Because an extensive theoretical description of barrier-limited transport is a major challenge, detailed computer models currently remain the most accurate nonexperimental methods for investigating ion transport in the barrier-limited regime. Given how sensitively the permeation regime depends on the pore size, our results suggest that in addition to advances in fabrication, accurate theoretical interpretation of measured transport data is vital to harnessing the unique features of barrier-limited ionic and molecular transport in nanofluidic systems using nanoporous 2D materials

## 1 Introduction

Controllable aqueous ion transport is key to a wide variety of applications, including water purification,^1,2^ sensing,^3,4^ drug delivery,^5^ and neuromorphic computing.^6^ Our understanding of the mechanisms that underlie ion transport below the diffusive limit historically comes from biology, where it is central to a vast array of functions, ranging from neural signal transmission in the brain^7−9^ to sensory perception^10−14^ and muscle contraction.^15,16^ Basic insights are typically gained from biophysical studies of transport in protein ion channels.^17−19^ In fact, until relatively recently, protein ion channels in aqueous environment were the only entities capable of featuring the intricate interplay between local interactions (often sensitive to confinement variations at the deep subnanometer scale) requisite to enable barrier-limited transport. Thanks to the advances in fabrication techniques, however, nanoscale and subnanoscale pores in solid materials have emerged in the past decade as potentially promising in achieving ion permeation controlled by ion-specific local interactions.^2,18,20−24^ In addition, recent computational and theoretical studies have demonstrated that local barriers can enable ion-specific trapping of ions by the pores,^20,25,26^ mechanosensitive ion transport,^21,22,27^ as well a range of memory phenomena.^25,26^ At the basic level, nanoporous 2D materials in aqueous environment present systems structurally far less complex than their biological counterparts. Moreover, selective ion transport through subnanoporous 2D materials is often caused by the crown-like effects of complexation, suggesting an intriguing bridge between the fields of nanofluidics and coordination chemistry.^20,28^ Despite the potential promise in both the basic and applied areas, our ability to predict pore sizes and compositions capable of controllable transport marked by local barriers remains severely lacking. One particular limitation is in our ability to accurately predict the permeation regimes (diffusive or barrier-limited), especially in the case of electrically neutral pores, which feature dipolar interactions between permeants and the pore edge. The aim of this work is 2-fold: to shed light on the experimentally observable indications of the transition between diffusive and barrier-limited regimes of permeation through pores in 2D membranes and to determine the limits of applicability for the diffusive formalism widely used to interpret experimentally measured transport data. It is important to emphasize that the focus of this work is on water-dissociated ion transport through pores in 2D materials, because key barrier-limited transport phenomenology discussed here can be mimicked in significantly longer (and wider) channels in three-dimensional solids, despite being entirely diffusive. As an example, we consider aqueous alkali cation transport through electrically neutral triangular pores of varying sizes in hexagonal boron nitride (hBN) selected as a model material.

## 2 Methods

**Figure 1:**
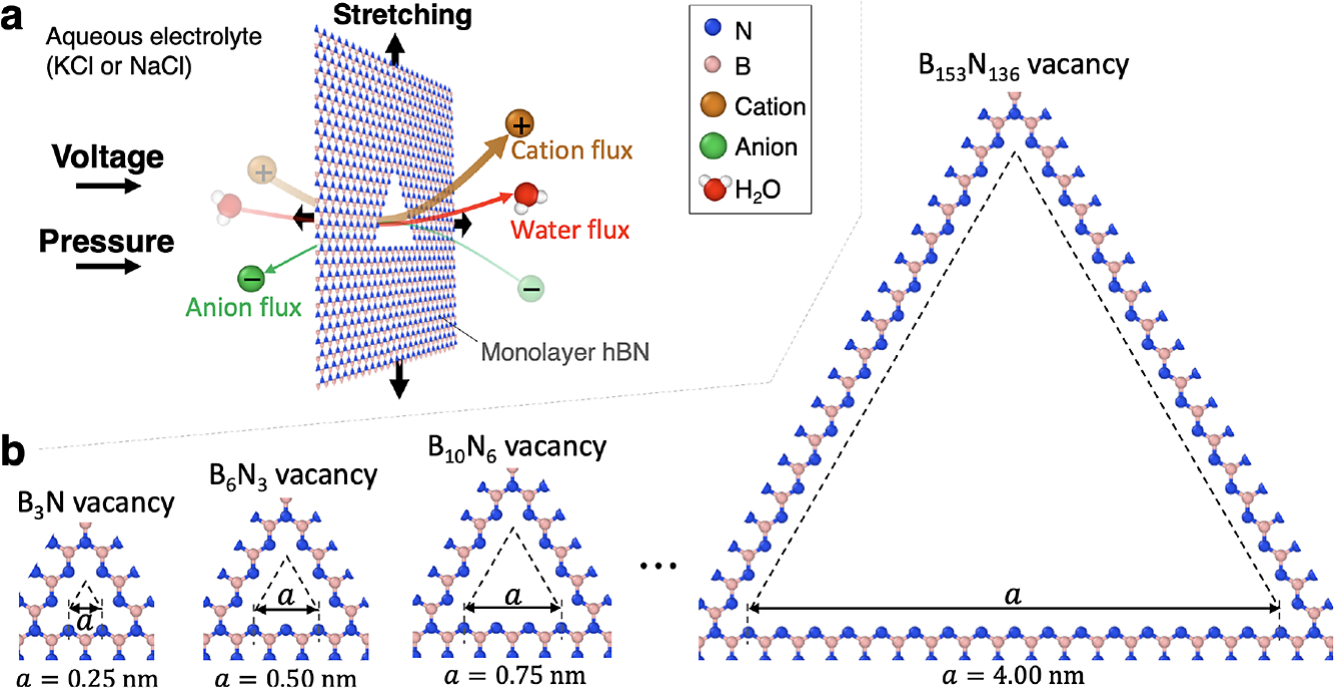
MD simulation setup for ion transport through various triangular pores in monolayer hBN. (a) Illustration of ion transport driven by voltage and/or pressure differences across a membrane subjected to stretching. (b) Triangular pores considered in this study.

Molecular dynamics simulations of ion transport through triangular nanopores in monolayer hBN were used in this work, carried out using GPU-accelerated GROMACS.^29,30^ Pore sizes ranging from 0.25 to 4.0 nm were investigated (see Figure 1). For pores up to 2.5 nm in effective size, the unstrained membrane dimensions were 5.97 × 5.97 nm. Larger pores were hosted by membranes sized 7.97 × 9.05 nm. In all cases, simulations were performed in a rectangular cell periodic in *XY Z*; cell size along *Z* was 6 nm. In simulations involving strained membranes, the initial structures were isotropically stretched in-plane (along with the corresponding cell dimensions) by the amounts stated in the main text. Throughout the simulated times, the edge atoms were tethered to their initial positions by isotropic harmonic restraints with the corresponding force constants of *k*_*X*_ = *k*_*Y*_ = *k*_*Z*_ = 16.667 N/m. All systems were solvated in aqueous salt species, as stated later in the text. The OPLS/AA framework^31^ was utilized for hBN,^32^ ions, rigid TIP4P water^33^ (with bonds maintained by the fourth order LINCS algorithm implemented in GROMACS^34^), including all nonbonded interactions. To ensure electrostatic neutrality of each pore structure, partial atomic charges of the nitrogen atoms at the pore edge were set to two-thirds of their bulk hBN counterparts, as developed elsewhere^32^ (see Table 1 therein for the complete bonded/nonbonded parameter set). The rest of the parameters were default OPLS-AA, as developed for GROMACS.

Coulomb interactions were resolved using the particle–particle–particle-mesh scheme with a 1.2 nm cutoff radius. The same cutoff was used for calculating the van der Waals interactions, as represented by the Lennard–Jones potential. Prior to production simulations, all systems underwent static energy minimization, followed by dynamic 5 ns-long relaxation in the NPT ensemble. In the latter, a semiisotropic barostat was applied along the *Z*-direction while keeping the *XY*-dimensions of the box constant. All production simulations were carried out in the NVT ensemble. Relaxation (NPT) and production (NVT) simulations were carried out using a time step of 1 and 2 fs, respectively.

Electrostatically driven ion transport simulations were performed under a constant electric field *E*_*z*_ applied perpendicularly to the membrane (along the *Z*-direction). Pressure-driven flow was induced by applying a constant force acting upon the solvent molecules in the form of artificial “gravity”: a force equal to *mg* was applied to the solution particles the *Z*-direction, where *m* and *g* is the particle mass and the artificial gravitational acceleration, respectively. Under this bias, the reported pressures are ρ*gh*, where ρ ≈ 1 g/cm^3^ is the average solution density and *h* ≈ 6 nm is the box height in the *Z*-direction. Each simulated data point for the reported ionic currents was obtained from an independent simulation. Each transport instance through the smallest pores (B_3_N multivacancy, *a* = 0.25 nm) was simulated for 500 ns, while transport through larger pores was simulated for 200 ns in each case. Ion currents were obtained from the cumulative ion fluxes, as described in detail in the Supporting Information. The output frequency of ion trajectory frames used to obtain the ion fluxes was once every 10 ps. Visualization of atomic configurations was performed using OVITO software.^35^

**Figure 2:**
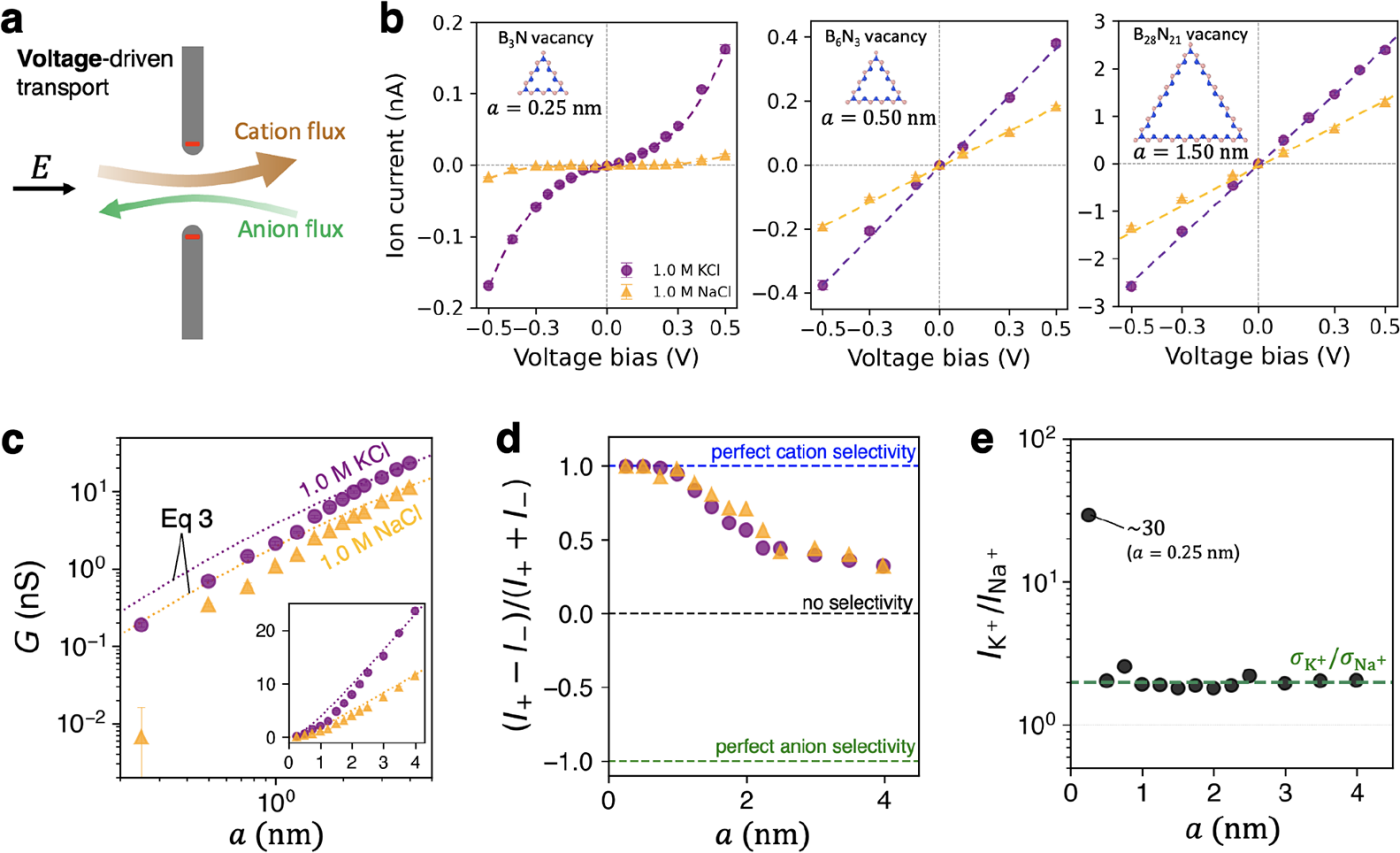
Voltage-driven ion transport for various pore sizes. (a) Sketch of voltage-driven transport. (b) Ion current–voltage curves for three different pore sizes: 0.25, 0.5, and 1.5 nm in 1 M KCl and NaCl solutions. The insets show the corresponding pore structures and sizes. (c) Log scale plot of ion conductance for various pore sizes. The dotted lines correspond to eq 3; the inset in (c) shows the same data plotted using the linear scale. (d) Anion–cation selectivity as a function of pore size. (e) K^+^/Na^+^ transport ratio for various pore sizes.

Estimates of bulk ionic conductance σ used to obtain the analytical curves in Figure 2c were obtained from separately performed 200 ns-long simulations of ion transport in a cubic simulation cell (4 nm-long side, periodic in *XYZ*) filled with bulk water-dissociated salt. The simulations were carried out at the temperature of 300 K and an electric field *E*_*z*_ = 0.05 V/nm. The bulk conductivity was then calculated from the resulting ion current *I* as , where *A* = 16 nm^2^ is the cross-sectional area of the simulation cell in the *XY*-plane. For 1 M KCl and NaCl, the values of σ are as stated in the text accompanying the results in Figure 2. For comparison, at 0.5 M, we obtained 5.544 ± 0.164 and 3.392 ± 0.221 S/m for KCl and NaCl, respectively.

## 3 Results and Discussion

### 3.1 Diffusive and Barrier-Limited Transport Modes

Electrophoretic ion transport through microscale and relatively wide nanoscale channels is often described as a diffusion-limited process accurately quantified by the classical diffusion theory. Within this framework, the corresponding total resistance can be approximated as the sum of the Maxwell–Hall access resistance^36,37^ and the channel resistance. The latter is typically associated with the ions’ translocation through the pore interior. Access resistance, on the other hand, can be viewed as a manifestation of ionic path convergence from bulk solvent into the channel lumen. For a circular pore, the resulting conductance for electrostatically driven ion transport is a simple inverse of the corresponding total resistance:
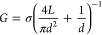
1where the two terms (from the left) correspond to the channel resistance and the access resistance, respectively; σ is the electrolyte conductivity in the suitable vicinity of the channel, and *L* and *d* are the pore length and diameter, respectively.^38,39^ For uncharged pores, the σ term is the bulk conductance. For pores carrying nonzero electric charge, separate values of anion and cation conductance values can in principle be introduced to account for the relative excess of counterions and scarcity of co-ions near the pore entrance.^40−42^ Ion transport described by eq 1 broadly describes continuum-like flow and in the past decades it has been widely used for pore size estimation based on the experimentally measured transport data. Despite its utility for large pores (5–10 nm in diameter), as shown later, eq 1 outright fails to describe the majority of ion transport in biology, as well as an emerging class of artificial subnm pores in various nanofluidics/nanoionics applications.

To describe ion transport through many protein ion channels with effective subnanoscale diameters (and, more generally, all subnanoscale pores), it is more appropriate to view the process as sequential “single-file” leaping of highly localized free energy barriers^17−19^ by the ions. This invokes transition state theory, wherein the local energy landscape along the transport coordinate essentially dictates the transport rate, often far below the diffusive limit. The corresponding mathematical description differs from the diffusive case through the introduction of barrier-dependent permeation probabilities. According to the Boltzmann statistics, the probability of overcoming an energy barrier is proportional to the corresponding Arrhenius exponent. In a sequential barrier-leaping process, the transport rate is then primarily determined by the highest energy barrier present in the corresponding free energy profile. The latter is known as the rate-limiting barrier and the corresponding ion conductance is given by an Arrhenius-type equation^2,21,43^:
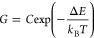
2where *C* determines the diffusive attempt frequency of barrier-leaping, Δ*E* is the rate-limiting free energy barrier, *k*_B_ is the Boltzmann constant, and *T* is the system temperature. Since Δ*E* is a quantity specific to a given pore-permeant pair, this equation can describe permeation selectivity far beyond the mere differences in for example the permeants’ bulk conductivity. As suggested by eq 2, the change in Δ*E* results in an exponential change in permeability. As a result, in cases where Δ*E* changes in response to for example membrane stretching, Δ*E* modifications of order few *k*_B_*T* can have a significant impact on the ion current.^21−23,27,44,45^ In addition, several unique ion transport phenomena arise in the barrier-limited transport regime, such as selective trapping,^21,25,26^ memristive transport,^25^ and synaptic-like phenomena^26^ resulting from collective effects in pore arrays featuring strongly barrier-limited pores.

Here we consider examples of simulated ion transport in the diffusive and barrier-limited regimes. Shown in Figure 1a is a sketch of the voltage- and pressure-driven ion transport through a porous 2D membrane under isotropic tensile strain. The results of pressure-driven simulations are presented in the Supporting Information, while the results for electrostatically driven transport are presented in the main text.

Aqueous KCl and NaCl were considered as test salts. Pore sizes ranged from 0.25 to 4.0 nm (see Figure 1b for pore size definition and several pore structure examples), corresponding to strictly local barrier-limited and diffusive regimes, respectively. All of the shown triangular pores are nitrogen-terminated, causing dipolar electrostatics at the pore edge, with the negative dipole component oriented toward the pore interior, similar to crown ethers in graphene and other crown-like structures.^20,44^ The justification for our selection of material and pore type is 2-fold. First, nitrogen-terminated triangular multivacancies have been previously reported as stable^46−49^ and a highly controllable approach to fabrication was recently demonstrated.^50,51^ Second, the presence of a donor–acceptor atomic pair within hBN’s unit cell suggests that in principle no chemical functionalization beyond pore size is required to engineer the local pore electrostatics.^27^

Electrostatically driven ion transport is sketched in Figure 2a, with cations and anions permeating in opposite directions, each species contributing to a net current along the electric field vector. The dipolar electrostatics featuring negative charges of the nitrogen atoms lining the pore edge is expected to induce a degree of cation selectivity, depending on the pore size. Shown in Figure 2b is the current–voltage response for pores of three different sizes. For the smallest pore size (B_3_N vacancy, *a* = 0.25 nm), the current–voltage curves exhibit highly nonlinear behavior. For larger pores (*a* = 0.5 nm and *a* = 1.5 nm), however, the current–voltage curves are nearly linear. Granted, the current–voltage curve linearity is far from a reliable criterion on the transport type (diffusive or barrier-limited), because in the case of porous 2D membranes the current–voltage nonlinearities can be readily caused by e.g., bias-induced ion crowding effects, as opposed to barriers imposed by ion-pore and ion–solvent interactions. At the same time, given the wide voltage bias range considered here, the clear transition from nonlinear to linear current–voltage dependence may serve as some indication of the presence of local barriers, i.e., the nonlinear and linear trends *may* correspond to barrier-limited and diffusive transport, respectively. In the considered case, the transition appears to take place when the pore size increases from *a* = 0.25 nm to *a* = 0.5 nm.

For a diffusive nanopore in the shape of equilateral triangle with side *a*, the total resistance is similar to the circular case in eq 1, as proposed earlier^52^:
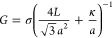
3where κ is a dimensionless constant of order 1. Shown in Figure 2c is the ion conductance as a function of *a*, plotted on the logarithmic scale, calculated in all cases as *I*/*V* at *V* = 0.3 V. The purple dots and orange triangles represent the MD data, while the dotted lines correspond to eq 3 with *L* ≈ 0.5 nm (see discussion accompanying supporting Figure S4 and also Figure 4 in our earlier work^21^), σ_bulk_ = 9.496 ± 0.270 S/m for 1 M KCl, and σ_bulk_ = 4.825 ± 0.183 S/m for 1 M NaCl. Our σ estimates were obtained from bulk MD simulations carried out separately for this work (see Methods for details). Equation 3 provides a fairly accurate conductance estimate for the largest considered pore (*a* = 4.0 nm), yielding *a* ≈ 3% error for 1 M of aqueous KCl and NaCl. However, eq 3 expectedly breaks down for smaller pores, at *a* = 0.25 nm overestimating the MD-simulated conductance by a factor of 2 and 30 for KCl and NaCl, respectively. This brings us to an important point: in experiments, eqs 1 and 3 have been widely used to roughly estimate pore sizes from measured ion currents, even though the applicability of these equations is severely limited for nm-scale pores, as evidenced by the data in Figure 2c. In fact, the effective pore size estimated by eq 3 is 0.68 nm for the 1.0 nm pore (∼32% error) while yielding an entirely nonsensical estimate of 0.04 nm for the 0.25 nm pore, as obtained for 1 M NaCl. More generally, the data in Figure 2c suggests that the discrepancies between simulated data and analytical predictions begin to exceed 25% for pores smaller than 1.25–1.5 nm (corresponding to *G* < 5 nS). Further discussion of the spatial limitations of the diffusive formalism is provided later in the text. These limitations are at a basic level and, although a comprehensive analytical description of barrier-limited transport is beyond the scope of this work, here we attempt to formulate a set of specific criteria for discriminating diffusive pores from those governed by local barriers. It is also worth noting that pore size estimate discrepancies discussed above cannot be reconciled by simply introducing the effects of surface charge. Although with nonzero pore charges selective transport readily emerges through the imbalance between co-ions and counterions, transport qualitatively remains diffusive.^3,53−55^ Therefore, in this work, we focus on electrically neutral pores featuring dipolar electrostatics and overall greater levels of steric confinement.

Cation–anion selectivity is an important aspect of ion transport. It is defined as *S*_*V*_ = (*I*^+^ – *I*^–^)/(*I*^+^ + *I*^–^), where *I*^+^ and *I*^–^ are the cation and anion current, respectively. Under this definition, a selectivity value of 1, −1, and 0 corresponds to perfect cation selectivity, perfect anion selectivity, and no selectivity, respectively. Shown in Figure 2*d* is the value of selectivity as a function of pore size: for pores with *a* = 0.25 and 0.5 nm, perfect cation selectivity is observed, while further increasing the pore size (*a* = 0.75 and 1.0 nm) expectedly causes selectivity to decrease (≈0.9 for both KCl and NaCl). For even larger pores, selectivity decreases further, reaching ≈ 0.3 at *a* = 4.0 nm. This finding is rather remarkable, given that these pores do not carry an electric charge and the selectivity arises only from relatively faint dipolar ion-pore interactions. Similar to the case of large charged pores mentioned above, however, the transport character remains close to diffusive, while selectivity is introduced via cation- and anion-specific values of σ. This suggests that cation–anion selectivity is generally unreliable to determine whether transport is diffusive or barrier-limited without detailed prior knowledge of pore sizes and local electrostatic compositions.

Compared with the anion–cation selectivity, however, *strong cation–cation* (or anion–anion) selectivity may in fact serve as a reasonable indicator of the presence of barriers caused by high confinement and short-range interactions. Shown in Figure 2e is the *I*_K^+^_/*I*_Na^+^_ current ratio for various pore sizes, as obtained from 1 M KCl and NaCl. With the exception of the smallest pore (*a* = 0.25 nm), all pores considered here exhibit negligible K^+^/Na^+^ selectivity, with *I*_K^+^_/*I*_Na^+^_ ≈ σ_K^+^_/σ_Na^+^_, where σ_K^+^_ and σ_Na^+^_ are the respective cationic components of the bulk conductivites. Notably, however, the smallest pore (*a* = 0.25 nm) exhibits high selectivity for K^+^ over Na^+^, with *I*_K^+^_/*I*_Na^+^_ ∼ 30. It is clear that the level of confinement featured by a pore of this size enables significant local contributions from the ion-pore van der Waals interactions, as well as cation-specific dehydration effects, known to be at the core of barrier-limited transport.^20,21,27^ Specifically in the context of the smallest pore considered in this work (B_3_N vacancy, *a* = 0.25 nm), K^+^ ions overcome a significantly lower barrier than Na^+^, as also shown previously.^56^ This result suggests that the cation–cation selectivity can indeed be a clear indicator of local barriers. For instance, the selectivity observed for monovalent cation transport through metal–organic frameworks with subnanometer-sized windows^57^ is likely to arise from the local barriers caused by extreme confinement and not merely diffusion through charged structures. We note once again that this type of selectivity as a possible indication of transport well below the diffusive prediction is generally only valid for the pores in 2D materials. For instance, significantly longer pores carrying surface charge can enable purely diffusive transport that occurs mostly along the pore surface^58,59^ (especially at low bulk salt concentrations), causing cation–cation selectivity.

### 3.2 Barrier-Limited Transport and Scaling of Mechanosensitivity

In addition to cation–cation (or anion–anion) transport selectivity, mechanosensitive response of ion flow can serve as a deeper indicator of transport governed by highly localized energy barriers. Furthermore, the degree of localization is directly reflected in the magnitude of mechanosensitivity. Mechanosensitive ion transport generally refers to the modulation of ion flow in response to externally applied mechanical stimuli. Although versions of broadly defined mechanically modulated ion transport have been recently demonstrated in artificial systems (e.g., in conical glass nanopores^60^ or in planar subnanoscale confinement^61^), here we use the relatively stringent biophysical definition of mechanosensitivity as *the response of ion transport to in-plane forces acting on the membrane and not the bias that causes those forces* (e.g., hydrostatic pressure perpendicular to the membrane).^62^ To avoid confusion, in this work we therefore refer to mechanosensitivity as the change in ion conductance only in response to pore dilation – regardless of what causes this dilation. The importance of this distinction from other effects that accompany mechanical deformation of the pores at the nanoscale (e.g., electrohydrodynamic ion flow^63,64^) will become apparent later in the text. It is important to note that for the small pores exhibiting highly mechanosensitive behavior, these effects are expected to be negligible (see Supporting Information for a detailed discussion of pressure-modulated ion transport).

Recently, mechanosensitivity under the specific definition above was computationally and theoretically predicted in a variety of subnanoporous 2D materials.^21,22,27,44,56^ These observations can be attributed to exclusively barrier-limited transport described in eq 2, where Δ*E* is reduced by a few *k*_B_*T* as a result of pore dilation of order few percent. It is worth noting that, although not immediately intuitive, slight pore enlargement can also *increase transport barriers*, resulting in a significant decrease in the ion flow and thus distinctly negative mechanosensitivity.^56^ However, for the purpose of this discussion, the direction of modulation (increasing or decreasing) in response to pore dilation is unimportant, as we focus on the absolute value of mechanosensitivity as a way of probing the sensitivity of ion flow to small changes in the effective pore diameter.

**Figure 3:**
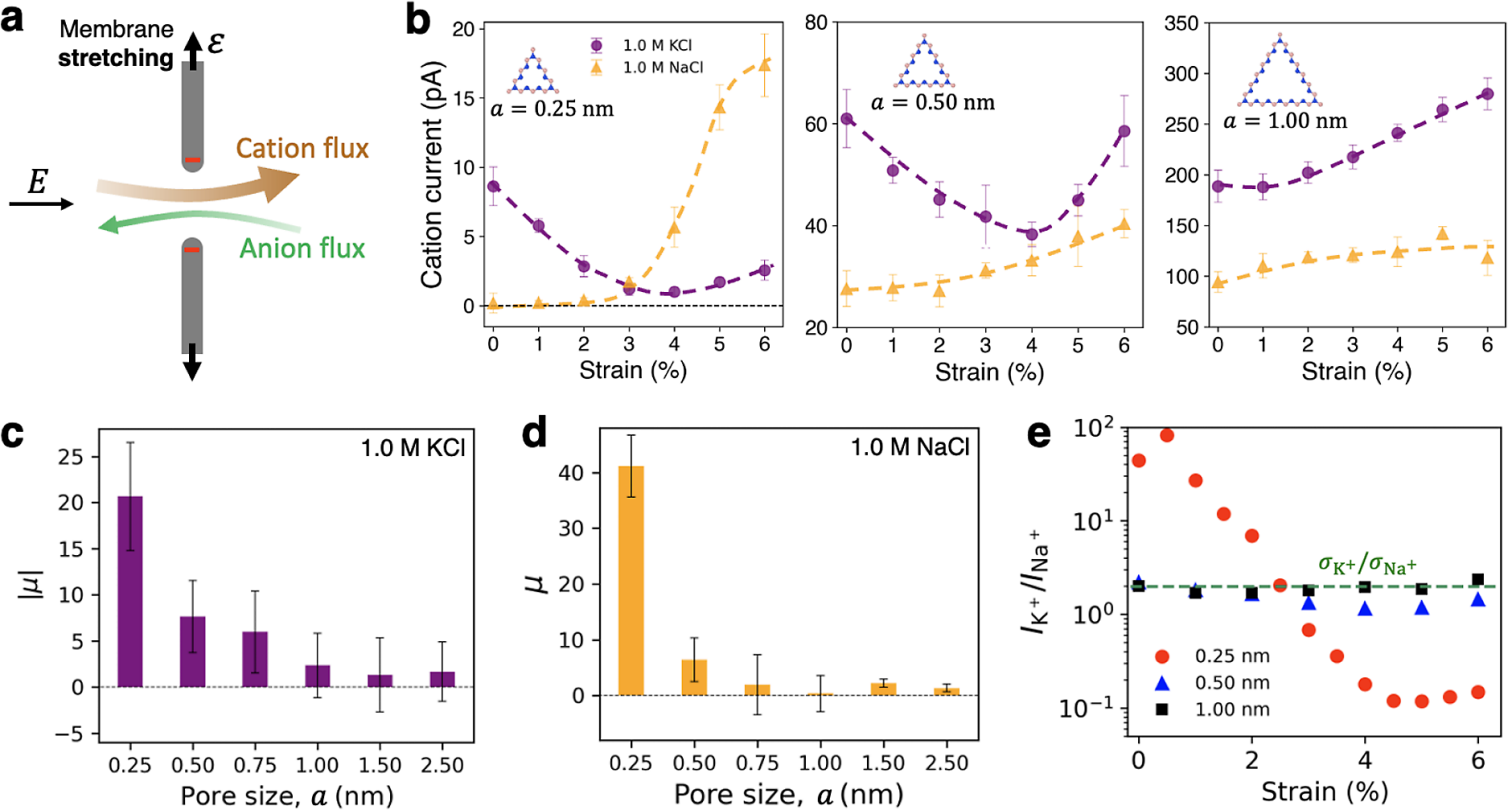
Scaling of mechanosensitivity with pore size. (a) Sketch of voltage-driven ion transport through a stretched membrane. (b) Ion current as a function of membrane strain for the pore sizes of 0.25, 0.50, and 1.00 nm, as simulated at the voltage bias of 0.1 V. (c) Mechanosensitivity as a function of unstrained pore size for K^+^ (c) and Na^+^(d). (e) K^+^/Na^+^ current ratio as a function of strain for three different pore sizes.

Let us define mechanosensitivity as the ratio between the relative change of the ion conductance and the relative dilation of a pore of radius *r*:
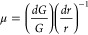
4which can be rewritten as
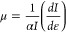
5where *I* is the simulated current, ε is the externally applied membrane strain, and α is the pore dilation factor of order 2, indicating how much “softer” the pore region is in relation to the rest of the pore-hosting membrane.^56^ Shown in Figure 3b are ionic currents vs membrane strain for several unstrained pore sizes, while the corresponding mechanosensitivity values (as calculated using eq 5) are shown in Figure 3c,d (note that for the K^+^ ions μ < 0^56^ and thus for clarity this panel shows – μ). As expected, the absolute value of μ depends on the cationic species and rapidly decreases with increasing unstrained pore size. It is also by far the highest at *a* = 0.25 nm for both cationic species. Similarly, as shown in Figure 3e, cation–cation selectivity stands out for the 0.25 nm-wide pore and decreases significantly for the larger pores. If we accept that mechanosensitive response is closely related to the permeation regime, let us briefly consider the limiting cases.

Under the definition given by eq 4, mechanosensitivity for the purely diffusive case in eq 1 is
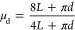
6which for a long pore (*L* ≫ *d*) of sufficiently large diameter reduces to μ_d_ = 2, or the exact value of mechanosensitivity of an ideal cylindrical ionic conductor with negligible access resistance. For wide pores in atomically thin membranes (*L* ≪ *d*), the access term dominates and mechanosensitivity decreases further to unity, as expected. Note that a similar estimate is valid for charged pores. Furthermore, if one assumes that the counterion component is proportional to the charge density at the pore edge,^40^ the corresponding mechanosensitivity component is of order −1. This in fact suggests the possibility of charged pores with zero mechanosensitivity, i.e., the decrease in counterion current counteracts the corresponding increase due to pore dilation.

In the limiting case of purely barrier-limited single-file transport, an estimate of mechanosensitivity is significantly more complex, because in order to be accurate it requires prior knowledge of Δ*E* and all of its contributing components as a function of pore size in eq 2. Here we limit our discussion to qualitative scaling observations, which become possible under suitable basic assumptions regarding the nature of Δ*E*. Based on eq 2, mechanosensitivity becomes
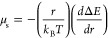
7Given that Δ*E* is fundamentally electrostatic, including the contributions from first-order Coulomb interactions for charged pores (∝ 1/*r*) and the higher orders for dipolar interactions and beyond, let us define Δ*E* = ∑ Δ*E*_*n*_, where Δ*E*_*n*_ = *a*_*n*_*r*^–*n*^ and *a*_*n*_ is the appropriate constant for the *n*th interaction term; the summation is over all of the free energy components (excluding the entropic terms for simplicity). eq 7 then yields
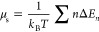
8which is merely a *k*_B_*T*-normalized sum of the free energy components, each *multiplied by the corresponding interaction order*. Qualitatively, this type of scaling is no different from the diffusive case, where mechanosensitivity is determined by the power of pore size in the conductance expression. For the barrier-limited case of sufficiently small pores, the pore size serves as an implicit “selector” of the interactions dominating the current-strain response. In addition, eq 8 illustrates that for example the van der Waals interactions become critically important in the estimates of mechanosensitivity due to e.g., *n* = 6 and *n* = 12 in the Lennard-Jones approximation for each interacting atom-ion pair. Given eq 8, it is not surprising that even with Δ*E*_*n*_ of order *k*_B_*T* it is possible for mechanosensitivity to reach levels of order 20–40 (see the results for *a* = 0.25 nm in Figure 3c,d), consistent with previous reports.^21,22,27^ It should then be clear that observations of mechanosensitivity an order of magnitude or higher above the diffusive estimates presented earlier can be a reliable indication of transport governed by local barriers.

### 3.3 Diffusion Formalism and Its Applicability Limits

A major challenge in describing ion transport over a wide range of pore sizes is to formulate a consistent theoretical description that helps interpret experimental data. In particular, such a description should not only reconcile the seemingly disparate limiting cases given by eqs 1 and 2, but also describe the transition between these limits. As mentioned above, developing such a theory is beyond the scope of this work. We note that at the most basic level, we expect any self-consistent theoretical description that is not rooted in merely fitting experimentally observed ionic currents to require transport barrier estimates. Obtaining the latter is notoriously challenging in experiments,^65,66^ while a relatively accurate alternative is likely to rely on numerical simulations (e.g., in the form of MD or density functional theory). The MD-based route, however, is computationally close to direct transport simulations, which take barriers into account implicitly. Ultimately, the path toward a detailed understanding of ion transport through subnanoscale pores in 2D materials remains open. In the meantime, here we employ all-atom MD simulations to investigate permeation regimes involving a strong barrier-governed component. In addition, we provide a relatively robust estimate of the critical pore size, below which eq 1 and similar expressions begin to fail.

**Figure 4:**
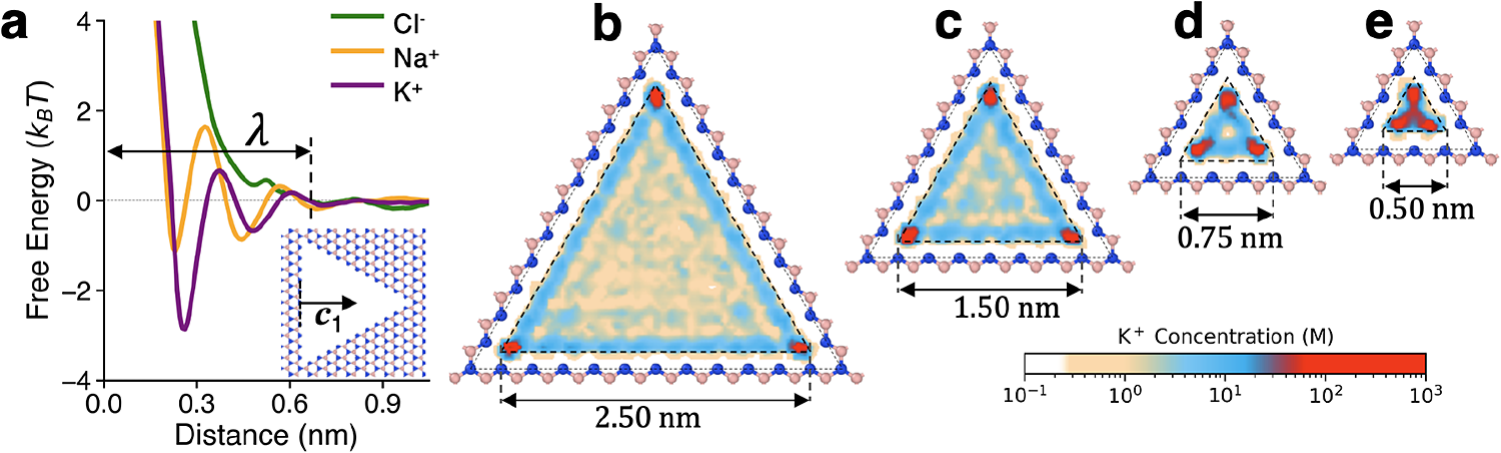
In-plane free energy curves and K^+^ populations inside the pore. In-plane ion-wall distance and the corresponding free energy (a) along vector  shown in the inset. In-plane distributions of the effective probability of finding a cation within the pore plane for various pore sizes (b–e); the probability density is shown in the units of concentration.

Between the strictly diffusive and barrier-limited regimes, both the local ion densities and the permeation rates near the pore edge differ from those toward the pore center. This expectation is no different from ion transport through wide charged nanopores previously considered in the literature,^67^ except here the pores are significantly shorter and, given overall electrically neutral pores, the interactions here are generally shorter-range with a weaker dependence on the local charge screening. Nevertheless, a direct relationship between the spatial extension of the ion-pore interactions to the limit of applicability of the diffusive formalism is possible here in a similar manner. To demonstrate it, we calculate the in-plane free energy profile as a function of distance between the ion and the pore edge along the vector , as shown in the inset of Figure 4a.

The results for K^+^, Na^+^, and Cl^–^ ions are shown in Figure 4a, indicating energy minima located at 0.26 and 0.23 nm for K^+^ and Na^+^, respectively. Given the well depth for K^+^, narrow regions of ion accumulation are expected and confirmed in Figure 4b–e. Interestingly, a comprehensive evaluation of the in-plane energy profile yields a qualitative view far beyond mere accessibility regions dictated by the simplistic steric exclusion based on the van der Waals radii. We can now introduce a spatial threshold λ such that |Δ*E*|≪ *k*_B_*T* at distances ≥ λ from the edge. Based on the data in Figure 4a, we estimate λ ≈ 0.62 nm for both K^+^ and Na^+^ (although in general λ is expected to be ion-dependent). As defined, λ is equivalent to roughly twice the Debye screening length in the case of large charged pores, where double-layer overlap underlies the transition from bulk-like transport regime (*R* > λ, where *R* is the radius of a cylindrical pore) to that governed by the effects of charged pore surface (*R* < λ).^67^ In the same manner, the diffusive formalism in our case is expected to fail when the potential energy curves overlap near the pore center. For a pore in the shape of an equilateral triangle, this condition is met when  nm (*a*_0_ = 0.25 nm is the lattice constant of hBN) when *a* is defined as shown in Figure 1b. For circular pore geometries, assuming similar λ, this limit in the form of pore diameter is 2λ = 1.24 nm. We note that the thresholds proposed above do not predict the spatial scale at which high mechanosensitivity may occur. Given the data in Figure 4a, it is in fact expected to occur at scales < λ, because, as discussed earlier, substantial mechanosensitivity requires appropriately high *d*|Δ*E*|/*dx*, where *x* is the distance from the pore edge. It is also worth noting that the ionic flow and the local ion population in the pore undergo significant changes as the pore size decreases below the estimated threshold. As shown in Figure 4b–e for the ion population, the bulk-like region (in yellowish color toward the pore center) shrinks rapidly, while local charge accumulation starts to dominate. This includes the three single-file transport paths in the pore corners featuring highly localized electric fields (red spots in Figure 4b–e). Notably, when the effective pore size decreases below *a* = 0.75 nm (Figure 4d), the three corner paths merge into one and the pore becomes crown-like, as described previously in substantial detail.^20,27,56^ This transport regime is strictly barrier-limited, e.g., in the case of ion-trapping pores naturally corresponding to a pore size that causes an overlap of the free energy minima locations in Figure 4a.

## 4 Conclusions

We have presented the results of MD-simulated ion transport through 2D membranes hosting dipolar pores of various sizes with the aim of exploring the transition between diffusive and barrier-limited transport. We have identified high cation–cation (and anion–anion) selectivity and mechanosensitivity as reasonable indicators of barrier-limited transport. As shown in the Supporting Information, we also demonstrated that in the case of subnanoporous membranes biased by hydrostatic pressure or a combination of hydrostatic pressure and electrostatic bias, streaming contributions to overall ionic currents should be negligible. This finding suggests that purely mechanosensitive effects (arising from pore dilation) are expected to be observable in experiments without being masked by the effects contributed by the mechanisms that cause membrane stretching (e.g., hydrostatic pressure).

Our results broadly suggest that the diffusive transport formalism widely used to estimate pore sizes based on the ionic currents is expected to break down for effective pore sizes below the orders of 2 and 1 nm for triangular and circular pores, respectively. Therefore, utmost care should be exercised when interpreting ion transport data obtained for subnanoporous 2D membranes. More generally, detailed knowledge of the local barriers is required to accurately describe barrier-limited transport and thus reliable control over fabricating and characterizing pore structures is required in terms of geometries and atomic composition. Overall, our results underscore the importance of precise subnm pore fabrication and a careful approach to interpreting measured transport data before the unique features of barrier-limited transport can be effectively utilized for nanofluidic/nanoionic applications.

## Supporting Information Available

Additional data and discussion of the effects of hydrostatic pressure on ion transport; additional data on mechanosensitive response; details on pore length definition, methodology and data processing (PDF)

## References

[ref1] Humplik T.; Lee J.; O’Hern S.; Fellman B.; Baig M.; Hassan S.; Atieh M.; Rahman F.; Laoui T.; Karnik R.; Wang E. Nanostructured materials for water desalination. Nanotechnology 2011, 22, 292001 10.1088/0957-4484/22/29/292001. 21680966

[ref2] Epsztein R.; DuChanois R. M.; Ritt C. L.; Noy A.; Elimelech M. Towards single-species selectivity of membranes with subnanometre pores. Nat. Nanotechnol. 2020, 15, 426–436. 10.1038/s41565-020-0713-6. 32533116

[ref3] Höltzel A.; Tallarek U. Ionic conductance of nanopores in microscale analysis systems: where microfluidics meets nanofluidics. J. Sep. Sci. 2007, 30, 1398–1419. 10.1002/jssc.200600427. 17623420

[ref4] Schoch R. B.; Han J.; Renaud P. Transport phenomena in nanofluidics. Rev. Mod. Phys. 2008, 80, 839–883. 10.1103/RevModPhys.80.839.

[ref5] Duan R.; Xia F.; Jiang L. Constructing Tunable Nanopores and Their Application in Drug Delivery. ACS Nano 2013, 7, 8344–8349. 10.1021/nn405092w. 24143925

[ref6] Noy A.; Darling S. B. Nanofluidic computing makes a splash. Science 2023, 379, 143–144. 10.1126/science.adf6400. 36634195

[ref7] Morris C. E.; Sigurdson W. J. Stretch-inactivated ion channels coexist with stretch-activated ion channels. Science 1989, 243, 807–809. 10.1126/science.2536958. 2536958

[ref8] Raha A.; Wu Y.; Zhong L.; Raveenthiran J.; Hong M.; Taiyab A.; Wang L.; Wang B.; Geng F. Exploring Piezo1, Piezo2, and TMEM150C in human brain tissues and their correlation with brain biomechanical characteristics. Mol. Brain 2023, 16, 83 10.1186/s13041-023-01071-5. 38124148 PMC10731887

[ref9] Kandel E. R.; Schwartz J. H.; Jessell T. M.; Siegelbaum S.; Hudspeth A. J.; Mack S., Principles of neural science; McGraw-hill: New York, 2000; Vol. 4.

[ref10] Dance A. The quest to decipher how the body’s cells sense touch. Nature 2020, 577, 158–161. 10.1038/d41586-019-03955-w. 31913365

[ref11] Handler A.; Ginty D. D. The mechanosensory neurons of touch and their mechanisms of activation. Nat. Rev. Neurosci. 2021, 22, 521–537. 10.1038/s41583-021-00489-x. 34312536 PMC8485761

[ref12] Yang W.; Lin L.; Hu S.; Jiang B.; Yang R.; Yu W.; Tang J.; Zhao D.; Gu Y.; Jin M.; et al. Expression patterns of mechanosensitive ion channel PIEZOs in irreversible pulpitis. BMC Oral Health 2024, 24, 465 10.1186/s12903-024-04209-6. 38627713 PMC11022356

[ref13] McPherson D. R. Sensory hair cells: an introduction to structure and physiology. Integrative and comparative biology 2018, 58, 282–300. 10.1093/icb/icy064. 29917041 PMC6104712

[ref14] Gillespie P. G.; Müller U. Mechanotransduction by hair cells: models, molecules, and mechanisms. Cell 2009, 139, 33–44. 10.1016/j.cell.2009.09.010. 19804752 PMC2888516

[ref15] Nielsen O. B.; Overgaard K. Ion gradients and contractility in skeletal muscle: the role of active Na+, K+ transport. Acta physiologica scandinavica 1996, 156, 247–256. 10.1046/j.1365-201X.1996.204000.x. 8729684

[ref16] McKenna M. J.; Renaud J.-M.; Ørtenblad N.; Overgaard K. A century of exercise physiology: effects of muscle contraction and exercise on skeletal muscle Na+, K+-ATPase, Na+ and K+ ions, and on plasma K+ concentration—historical developments. Eur. J. Appl. Physiol. 2024, 124, 681–751. 10.1007/s00421-023-05335-9. 38206444 PMC10879387

[ref17] Doyle D. A.; Cabral J. M.; Pfuetzner R. A.; Kuo A.; Gulbis J. M.; Cohen S. L.; Chait B. T.; MacKinnon R. The structure of the potassium channel: molecular basis of K+ conduction and selectivity. science 1998, 280, 69–77. 10.1126/science.280.5360.69. 9525859

[ref18] Noskov S. Y.; Bernèche S.; Roux B. Control of ion selectivity in potassium channels by electrostatic and dynamic properties of carbonyl ligands. Nature 2004, 431, 830–834. 10.1038/nature02943. 15483608

[ref19] Roux B. Ion Conduction and Selectivity in K+ Channels. Annu. Rev. Biophys. 2005, 34, 153–171. 10.1146/annurev.biophys.34.040204.144655. 15869387

[ref20] Smolyanitsky A.; Paulechka E.; Kroenlein K. Aqueous ion trapping and transport in graphene-embedded 18-crown-6 ether pores. ACS Nano 2018, 12, 6677–6684. 10.1021/acsnano.8b01692. 29940107

[ref21] Fang A.; Kroenlein K.; Riccardi D.; Smolyanitsky A. Highly mechanosensitive ion channels from graphene-embedded crown ethers. Nat. Mater. 2019, 18, 76–81. 10.1038/s41563-018-0220-4. 30478453

[ref22] Sahu S.; Elenewski J.; Rohmann C.; Zwolak M. Optimal transport and colossal ionic mechano-conductance in graphene crown ethers. Sci. Adv. 2019, 5, eaaw5478 10.1126/sciadv.aaw5478. 31309155 PMC6625819

[ref23] Thiruraman J. P.; Masih Das P.; Drndić M. Stochastic Ionic Transport in Single Atomic Zero-Dimensional Pores. ACS Nano 2020, 14, 11831–11845. 10.1021/acsnano.0c04716. 32790336 PMC9615559

[ref24] Barabash M. L.; Gibby W. A. T.; Guardiani C.; Luchinsky D. G.; Luan B.; Smolyanitsky A.; McClintock P. V. E. Field-Dependent Dehydration and Optimal Ionic Escape Paths for C2N Membranes. J. Phys. Chem. B 2021, 125, 7044–7059. 10.1021/acs.jpcb.1c03255. 34115497 PMC8279548

[ref25] Noh Y.; Smolyanitsky A. Memristive Response and Capacitive Spiking in Aqueous Ion Transport through Two-Dimensional Nanopore Arrays. J. Phys. Chem. Lett. 2024, 15, 665–670. 10.1021/acs.jpclett.3c03156. 38206569 PMC10947333

[ref26] Noh Y.; Smolyanitsky A. Synaptic-like plasticity in 2D nanofluidic memristor from competitive bicationic transport. Sci. Adv. 2024, 10, eadr1531 10.1126/sciadv.adr1531. 39504376 PMC11540034

[ref27] Fang A.; Kroenlein K.; Smolyanitsky A. Mechanosensitive ion permeation across subnanoporous MoS2 monolayers. J. Phys. Chem. C 2019, 123, 3588–3593. 10.1021/acs.jpcc.8b11224.

[ref28] Guo J.; Lee J.; Contescu C. I.; Gallego N. C.; Pantelides S. T.; Pennycook S. J.; Moyer B. A.; Chisholm M. F. Crown ethers in graphene. *Nature*. Communications 2014, 5, 5389 10.1038/ncomms6389. 25391367

[ref29] Abraham M. J.; Murtola T.; Schulz R.; Páll S.; Smith J. C.; Hess B.; Lindahl E. GROMACS: High performance molecular simulations through multi-level parallelism from laptops to supercomputers. SoftwareX 2015, 1, 19–25. 10.1016/j.softx.2015.06.001.

[ref30] Páll S.; Zhmurov A.; Bauer P.; Abraham M.; Lundborg M.; Gray A.; Hess B.; Lindahl E. Heterogeneous parallelization and acceleration of molecular dynamics simulations in GROMACS. J. Chem. Phys. 2020, 153, 134110 10.1063/5.0018516. 33032406

[ref31] Jorgensen W. L.; Maxwell D. S.; Tirado-Rives J. Development and testing of the OPLS all-atom force field on conformational energetics and properties of organic liquids. J. Am. Chem. Soc. 1996, 118, 11225–11236. 10.1021/ja9621760.

[ref32] Govind Rajan A.; Strano M. S.; Blankschtein D. Ab initio molecular dynamics and lattice dynamics-based force field for modeling hexagonal boron nitride in mechanical and interfacial applications. J. Phys. Chem. Lett. 2018, 9, 1584–1591. 10.1021/acs.jpclett.7b03443. 29528646

[ref33] Jorgensen W. L.; Chandrasekhar J.; Madura J. D.; Impey R. W.; Klein M. L. Comparison of simple potential functions for simulating liquid water. J. Chem. Phys. 1983, 79, 926–935. 10.1063/1.445869.

[ref34] Hess B.; Bekker H.; Berendsen H. J. C.; Fraaije J. G. E. M. LINCS: A linear constraint solver for molecular simulations. J. Comput. Chem. 1997, 18, 1463–1472. 10.1002/(SICI)1096-987X(199709)18:12<1463::AID-JCC4>3.0.CO;2-H.

[ref35] Stukowski A. Visualization and analysis of atomistic simulation data with OVITO–the Open Visualization Tool. Modell. Simul. Mater. Sci. Eng. 2010, 18, 015012 10.1088/0965-0393/18/1/015012.

[ref36] Hall J. E. Access resistance of a small circular pore. J. Gen. Physiol. 1975, 66, 531–532. 10.1085/jgp.66.4.531. 1181379 PMC2226214

[ref37] Maxwell J. C. A treatise on electricity and magnetism; Clarendon Press: Oxford, 1873; Vol. 1.

[ref38] Kowalczyk S. W.; Grosberg A. Y.; Rabin Y.; Dekker C. Modeling the conductance and DNA blockade of solid-state nanopores. Nanotechnology 2011, 22, 315101 10.1088/0957-4484/22/31/315101. 21730759

[ref39] Suk M. E.; Aluru N. Ion transport in sub-5-nm graphene nanopores. J. Chem. Phys. 2014, 140, 084707 10.1063/1.4866643. 24588191

[ref40] Stein D.; Kruithof M.; Dekker C. Surface-charge-governed ion transport in nanofluidic channels. Phys. Rev. Lett. 2004, 93, 035901 10.1103/PhysRevLett.93.035901. 15323836

[ref41] Noh Y.; Aluru N. R. Ion transport in electrically imperfect nanopores. ACS Nano 2020, 14, 10518–10526. 10.1021/acsnano.0c04453. 32806038

[ref42] Bocquet L.; Charlaix E. Nanofluidics, from bulk to interfaces. Chem. Soc. Rev. 2010, 39, 1073–1095. 10.1039/B909366B. 20179826

[ref43] Zwolinski B. J.; Eyring H.; Reese C. E. Diffusion and Membrane Permeability. J. Phys. Chem. 1949, 53, 1426–1453. 10.1021/j150474a012.

[ref44] Smolyanitsky A.; Fang A.; Kazakov A. F.; Paulechka E. Ion transport across solid-state ion channels perturbed by directed strain. Nanoscale 2020, 12, 10328–10334. 10.1039/D0NR01858A. 32367087

[ref45] Sahu S.; Zwolak M. Diffusion limitations and translocation barriers in atomically thin biomimetic pores. Entropy 2020, 22, 1326 10.3390/e22111326. 33287091 PMC7712548

[ref46] Jin C.; Lin F.; Suenaga K.; Iijima S. Fabrication of a Freestanding Boron Nitride Single Layer and Its Defect Assignments. Phys. Rev. Lett. 2009, 102, 195505 10.1103/PhysRevLett.102.195505. 19518972

[ref47] Meyer J. C.; Chuvilin A.; Algara-Siller G.; Biskupek J.; Kaiser U. Selective Sputtering and Atomic Resolution Imaging of Atomically Thin Boron Nitride Membranes. Nano Lett. 2009, 9, 2683–2689. 10.1021/nl9011497. 19480400

[ref48] Alem N.; Erni R.; Kisielowski C.; Rossell M. D.; Gannett W.; Zettl A. Atomically thin hexagonal boron nitride probed by ultrahigh-resolution transmission electron microscopy. Phys. Rev. B 2009, 80, 155425 10.1103/PhysRevB.80.155425.

[ref49] Kotakoski J.; Jin C. H.; Lehtinen O.; Suenaga K.; Krasheninnikov A. V. Electron knock-on damage in hexagonal boron nitride monolayers. Phys. Rev. B 2010, 82, 113404 10.1103/PhysRevB.82.113404.

[ref50] Byrne D. O.; Raja A.; Noy A.; Ciston J.; Smolyanitsky A.; Allen F. I. Fabrication of Atomically Precise Nanopores in 2D Hexagonal Boron Nitride Using Electron and Ion Beam Microscopes. Microsc. Microanal. 2023, 29, 1375–1376. 10.1093/micmic/ozad067.707.

[ref51] Byrne D. O.; Allen F. I. Atomic Engineering of Triangular Nanopores in Monolayer hBN: A Decoupled Seeding and Growth Approach. ACS Appl. Nano Mater. 2025, 8, 4565–4572. 10.1021/acsanm.4c06998.

[ref52] Liu K.; Lihter M.; Sarathy A.; Caneva S.; Qiu H.; Deiana D.; Tileli V.; Alexander D. T.; Hofmann S.; Dumcenco D.; et al. Geometrical effect in 2D nanopores. Nano Lett. 2017, 17, 4223–4230. 10.1021/acs.nanolett.7b01091. 28592108

[ref53] Kirby B. J.; Hasselbrink E. F. Jr Zeta potential of microfluidic substrates: 1. Theory, experimental techniques, and effects on separations. Electrophoresis 2004, 25, 187–202. 10.1002/elps.200305754. 14743473

[ref54] Smolyanitsky A.; Saraniti M. Silicon nanopores as bioelectronic devices: a simulation study. Journal of Computational Electronics 2009, 8, 90–97. 10.1007/s10825-009-0275-1.

[ref55] Joshi P.; Smolyanitsky A.; Petrossian L.; Goryll M.; Saraniti M.; Thornton T. J. Field effect modulation of ionic conductance of cylindrical silicon-on-insulator nanopore array. J. Appl. Phys. 2010, 107, 054701 10.1063/1.3298468.

[ref56] Noh Y.; Smolyanitsky A. Stretch-inactivated ion transport through subnanoporous two-dimensional membranes. Phys. Rev. Mater. 2024, 8, L103001 10.1103/PhysRevMaterials.8.L103001.

[ref57] Zhang H.; Hou J.; Hu Y.; Wang P.; Ou R.; Jiang L.; Liu J. Z.; Freeman B. D.; Hill A. J.; Wang H. Ultrafast selective transport of alkali metal ions in metal organic frameworks with subnanometer pores. Sci. Adv. 2018, 4, eaaq0066 10.1126/sciadv.aaq0066. 29487910 PMC5817922

[ref58] Ritt C. L.; de Souza J. P.; Barsukov M. G.; Yosinski S.; Bazant M. Z.; Reed M. A.; Elimelech M. Thermodynamics of Charge Regulation during Ion Transport through Silica Nanochannels. ACS Nano 2022, 16, 15249–15260. 10.1021/acsnano.2c06633. 36075111

[ref59] Bush S. N.; Ken J. S.; Martin C. R. The Ionic Composition and Chemistry of Nanopore-Confined Solutions. ACS Nano 2022, 16, 8338–8346. 10.1021/acsnano.2c02597. 35486898

[ref60] Jubin L.; Poggioli A.; Siria A.; Bocquet L. Dramatic pressure-sensitive ion conduction in conical nanopores. Proc. Natl. Acad. Sci. U. S. A. 2018, 115, 4063–4068. 10.1073/pnas.1721987115. 29610303 PMC5910861

[ref61] Mouterde T.; Keerthi A.; Poggioli A.; Dar S. A.; Siria A.; Geim A. K.; Bocquet L.; Radha B. Molecular streaming and its voltage control in ångström-scale channels. Nature 2019, 567, 87–90. 10.1038/s41586-019-0961-5. 30842639

[ref62] Martinac B. Mechanosensitive ion channels: molecules of mechanotransduction. J. Cell Sci. 2004, 117, 2449–2460. 10.1242/jcs.01232. 15159450

[ref63] Jiang X.; Zhao C.; Noh Y.; Xu Y.; Chen Y.; Chen F.; Ma L.; Ren W.; Aluru N. R.; Feng J. Nonlinear electrohydrodynamic ion transport in graphene nanopores. Sci. Adv. 2022, 8, eabj2510 10.1126/sciadv.abj2510. 35030026 PMC8759738

[ref64] Zhang X.; Tu B.; Cao Z.; Fang M.; Zhang G.; Yang J.; Ying Y.; Sun Z.; Hou J.; Fang Q.; et al. Anomalous Mechanical and Electrical Interplay in a Covalent Organic Framework Monolayer Membrane. J. Am. Chem. Soc. 2023, 145, 17786–17794. 10.1021/jacs.3c04655. 37537964

[ref65] Shefer I.; Lopez K.; Straub A. P.; Epsztein R. Applying Transition-State Theory to Explore Transport and Selectivity in Salt-Rejecting Membranes: A Critical Review. Environ. Sci. Technol. 2022, 56, 7467–7483. 10.1021/acs.est.2c00912. 35549171

[ref66] Schwindt N. S.; Avidar M.; Epsztein R.; Straub A. P.; Shirts M. R. Interpreting effective energy barriers to membrane permeation in terms of a heterogeneous energy landscape. J. Membr. Sci. 2024, 712, 123233 10.1016/j.memsci.2024.123233.

[ref67] Probstein R. F. Physicochemical Hydrodynamics: An Introduction, 2nd ed.; Wiley: 2005.

