# Supplementary material for: Diffusive to Barrier-Limited Transition in the Aqueous Ion Transport through Nanoporous 2D Materials
Source: J Phys Chem B. 2025 May 6;129(19):4851–9. doi: 10.1021/acs.jpcb.5c00921 (PMC12086837; doi:10.1021/acs.jpcb.5c00921)
Supplement: Supplementary file 1 — jp5c00921_si_001.pdf [file jp5c00921_si_001.pdf]

Supporting Information for:

Diffusive to Barrier-limited Transition in the Aqueous Ion Transport Through Nanoporous 2D Materials

Yechan Noh

Department of Physics, University of Colorado Boulder, Boulder, CO 80309, USA

Applied Chemicals and Materials Division,

National Institute of Standards and Technology, Boulder, CO 80305, USA and

Department of Materials Science and Engineering,

University of California, Berkeley, Berkeley, CA 94720, USA

Alex Smolyanitsky

Applied Chemicals and Materials Division,

National Institute of Standards and Technology, Boulder, CO 80305, USA

PRESSURE-DRIVEN ION TRANSPORT

Mechanical modulation of ion transport through nanoporous structures can have multiple contributions, which are not straightforward to disentangle, especially in experiments. As discussed in the main text, under the biophysical definition of mechanosensitivity, the change in ion current is strictly due to pore dilation – regardless of what causes it. In a realistic nanofluidic system, however, if membrane stretching (and subsequent pore dilation) is achieved by applying hydrostatic pressure perpendicularly to membrane [1, 2], at least one additional mechanism emerges due to solvent permeation through the pores. For sufficiently large pores, if anions and cations are convectively dragged at different rates by the permeating solvent, a so-called streaming current is expected to arise, as sketched in Fig. S1a. The net current $(I^+ - I^-)$ is then a direct consequence of the permeation selectivity, reducing to zero for non-selective pores $(I^+ = I^-)$. If the streaming contribution to the measured current is comparable to that contributed by pore dilation, there is no straightforward way of separating the two phenomena.

Streaming ion transport has been widely studied in the context of *e.g.*, silica-based micro- and nanochannels. The existing theoretical descriptions are mainly centered around a Poisson-Boltzmann (PB) model with Poiseuille flow [3], as well as the Poisson-Nernst-Planck (PNP) model [4]. The PB-Poiseuille model provides a convenient semi-analytical approximation for the streaming current in one-dimensional channel geometries [3], while the PNP model is often solved numerically [4]. The continuum-level description in both models is based on the analysis of charge convection in the electrical double layer (EDL) formed near the channel surface. Importantly, the EDL structure and the corresponding convective velocity profiles described by these models do not include any explicit dependency on the salt type, essentially limiting the consideration to a generic anion *vs* cation flow problem. Although both models demonstrate good agreement with the experimentally observed streaming currents in channels with dimensions exceeding 70 nm [3, 4], predictive accuracy is far from guaranteed for channels only a few nanometers wide.

Here we briefly investigate streaming flow using all-atom MD simulations. For simplicity,

we define streaming conductance in accordance with continuum theories as follows: $G_{str} = I_P/P$, where P is the pressure bias and I_P is the streaming current. Shown in Figure S1b is the streaming current as a function of external pressure applied to the solvent along the Z-direction. For the considered pore sizes (a = 0.5 nm, 1.0 nm, and 1.5 nm), the current-pressure curves exhibit a fairly linear relationship within the tested range of pressure $|P| \leq 4.4$ kbar. For the smallest pore considered in the voltage-driven case above (a=0.5 nm), no ionic permeation was observed. In large pores, the streaming current exhibits negligible salt type dependency (see the right panel of Figure S1b), in agreement with behaviors described by the PB [3] and PNP [4] models. For smaller pores (*i.e.*, a = 0.5 nm and 1.0 nm), however, clear salt type dependence is observed. Specifically, the streaming current is higher in 1 M KCl solution compared to 1 M NaCl solution for both pores. This suggests the presence of short-range barriers affecting streaming transport.

Importantly, the simulated streaming currents remain at the picoampere scale even when the system is biased by pressure magnitudes of several kbar. Such pressure levels are several orders of magnitude higher than those typically used in experiments ($P \lesssim 10$ bar) [1]. Our use of high pressure (as compared to experiments), despite being significantly lower than pressure biases of ∼1000 kbar used in previous computational studies [2, 5, 6] is due to computational cost. More specifically, even state-of-the-art MD simulations performed at experimental pressure levels cannot yield statistically significant solvent or ion flux data within the simulated timescales of up to a few microseconds. Nonetheless, our results may still be experimentally relevant, if all ionic currents observed in simulations are appropriately rescaled for lower pressures, assuming that the linear dependence $I(P)$ in Fig. S1b remains valid at low pressures. Given the data in Fig. S1b, the rescaling argument suggests that for the streaming currents induced by experimentally relevant pressures should not exceed deep picoampere-order values, several orders of magnitude below the effects contributed by pore dilation. We also note that in our simulations of streaming phenomena the pore dilation itself is greatly underestimated due to the small pressurized area [7] (approximately 6 nm by 6 nm), *i.e.*, the product of pressure and area does not exceed a few nanonewtons. In experiments, however, the pressurized area of the membrane or substrate exceeds ∼ 100

μm^2, a few bar of pressure is expected to cause considerably greater deformation of the membrane [1]. Therefore, our streaming current simulations conveniently simulate the nearly pure pressure-driven ion current without the effect of pore enlargement, which is considered separately in the main text.

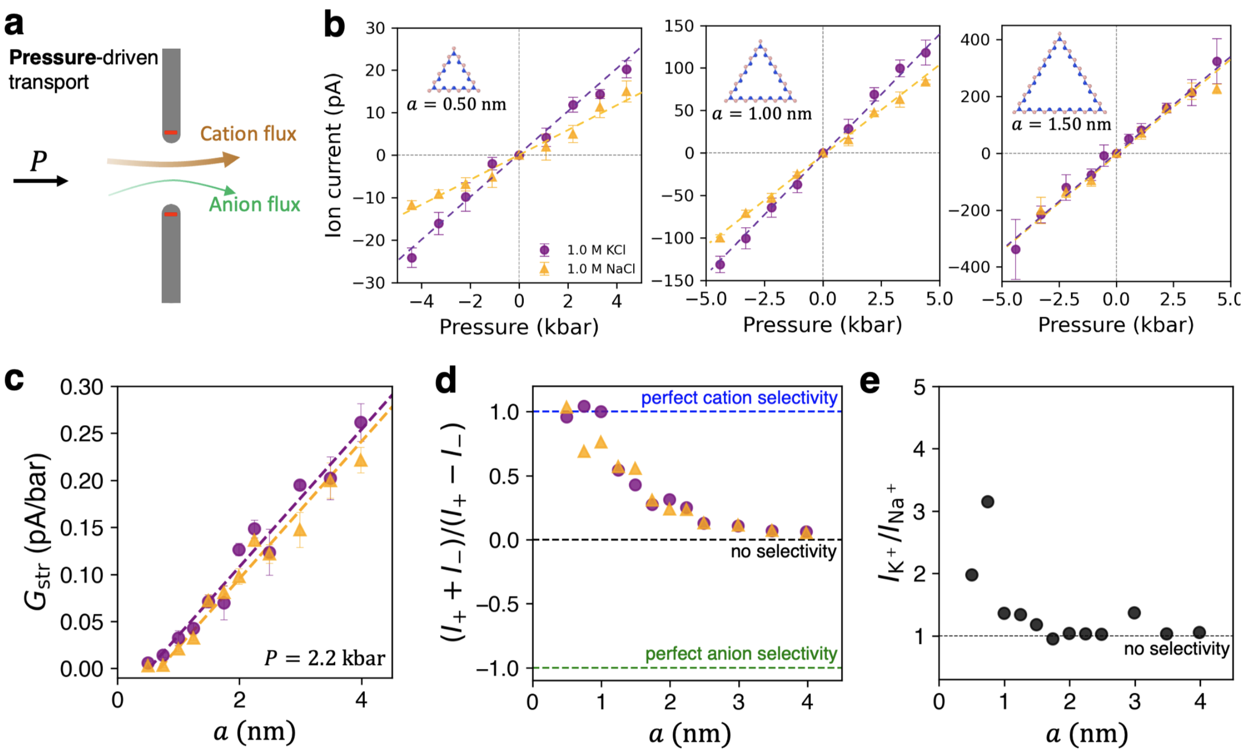

FIG. S1: **Pressure-driven ion transport for various pore sizes** (a) Sketch of pressure-driven transport. (b) Ion current-pressure curves for three different pore sizes: 0.5 nm, 1.0 nm, and 1.5 nm, from left to right, in 1 M KCl and NaCl solutions. The insets represent the respective pores. (c) Ion current normalized by pressure (2.2 kbar) for various pore sizes. (d) Selectivity between cations and anions as a function of pore size. (e) Ratio of K^+ transport over Na^+ transport for various pore sizes.

Shown in Fig. S1c is the streaming KCl and NaCl conductance ($G_{str} = I_P/P$ defined above), as obtained at $P = 2.2$ kbar for various pore sizes. For both salts, the dependence is nearly linear within the considered pore size range. This linear and not quadratic dependence on the effective pore diameter appears to be reasonable. Recall that streaming currents fundamentally arise from pore selectivity and the latter is a direct function of the EDL presence near the pore perimeter. Given that the pore perimeter is linear with respect to the effective pore size, this observation is overall consistent with our basic understanding

of streaming ion transport [3, 4]. We once again note that the streaming current induced by pressures within the typical experimental range of the pressure ($|P| \lesssim 10$ bar) remains small: $\lesssim 2$ pA for the largest pore we considered ($a = 4.0$ nm). Thus, the pure streaming component appears to be negligibly small compared to any electrostatically-driven currents simulated through the pores considered here.

VOLTAGE- AND PRESSURE-DRIVEN ION TRANSPORT

Ion transport in the presence of combined electrostatic and pressure biases, as well as potential coupling between them are considered next. Sketched in Figs S2a and S2b are the simulated scenarios with the electric field and pressure gradient co-directional and counter-directional, respectively. A co-directional combination induces co-directional forces acting upon cations, while the counter-directional arrangement aligns the two forces for anions. In the co-directional case, pressure is expected to enhance cation permeation (relative to the case without pressure) while reducing anion permeation. In the counter-directional arrangement, the effect is expected to be opposite. In addition, coupling between the effects of voltage and pressure is expected to arise [2], resulting from the effect of cross-membrane voltage on the EDL both at the pore edge and throughout the membrane [2]. When the two biases are applied co-directionally, for non-selective pores this coupling results in excess cationic flow, while in the counter-directional case this excess is anionic. As shown below, however, pore selectivity plays a key role in effectively modulating this coupling, depending on the direction of the pressure gradient relative to the direction of the voltage bias.

Including the coupling term, the total ion current can be expressed as a linear sum $I = I_V + I_P + I_{VP}$ [2], where I_V is the voltage-driven current in absence of pressure, I_P is the pressure-driven current in absence of voltage, and I_{VP} is the nonlinear voltage-pressure coupling term, which is nonzero only when both voltage and pressure biases are simultaneously present in the system. We considered the effect of the coupling term I_{VP} for several pore sizes by observing the excess current contributed by the addition of a pressure bias to an otherwise voltage-driven system: $\Delta I_P = I - I_{V,P=0} = I_P + I_{VP}$. The red triangles in Fig. S2c

represent ΔI_P as a function of pressure at a constant transmembrane voltage of 0.5V. In the same Figure, as a reference, we also plot the pressure-only case ($V = 0$) discussed in the previous section (and corresponding to $I_{VP} = 0$ or $\Delta I_P = I_P$). In the three panels of Fig. S2c (corresponding to the three pore sizes $a = 1.0$ nm, $a = 1.5$ nm, and $a = 2.5$ nm that yielded what we considered representative data), this reference is shown in gray circles. The difference between the two plots thus corresponds to the coupling term I_{VP}. As shown in Fig. S2c, $P < 0$ and $P > 0$ correspond to counter-directional and co-directional scenarios, respectively. Consistent with previously reported experimental results [2], the current-pressure curve exhibits a V-shaped trend that strengthens with increasing pore size. Consistent with the discussion above, this feature is due to transport enhancement by anions and cations at $P < 0$ and $P > 0$, respectively. However, for a pore of smaller size (*e.g.*, $a = 1.0$ nm in the leftmost panel of Fig. S2c), this enhancement ceases at $P < 0$ due to the anions being repelled by 1-nm-wide pore. This particular observation serves as a clear example of the effect of pore selectivity on systems with simultaneous pressure and voltage bias.

Shown in Fig. S2d is the cation/anion selectivity as a function of pressure, as calculated for several pore sizes. We observe increased cation selectivity in a co-directional pressure-voltage arrangement and increased anion selectivity in the counter-directional case, in qualitative agreement with the earlier discussion. Although simulated results suggest the geberal possibility of ion transport modulation by the presence of hydrostatic pressure itself (as opposed to the effect of pore dilation), for the considered pore sizes the pressure levels required to reach comparable contributions to measurable currents are far beyond experimentally achievable. As a rough quantitative estimate, Fig. S2e shows the excess streaming current ΔI_P normalized by pressure for various pore sizes. In particular, for the smallest considered pore ($a = 0.25$ nm), the voltage-pressure coupling effect is virtually nonexistent. Together with zero streaming currents through the same pores (when biased only by pressure, as mentioned in the previous section), it is reasonably clear that transport through pores at the deep sub-nm scales is affected by neither streaming effects, nor by voltage-pressure coupling. For larger pores, although $\Delta I_P/P$ increases, it remains at the the picoampere scale, which

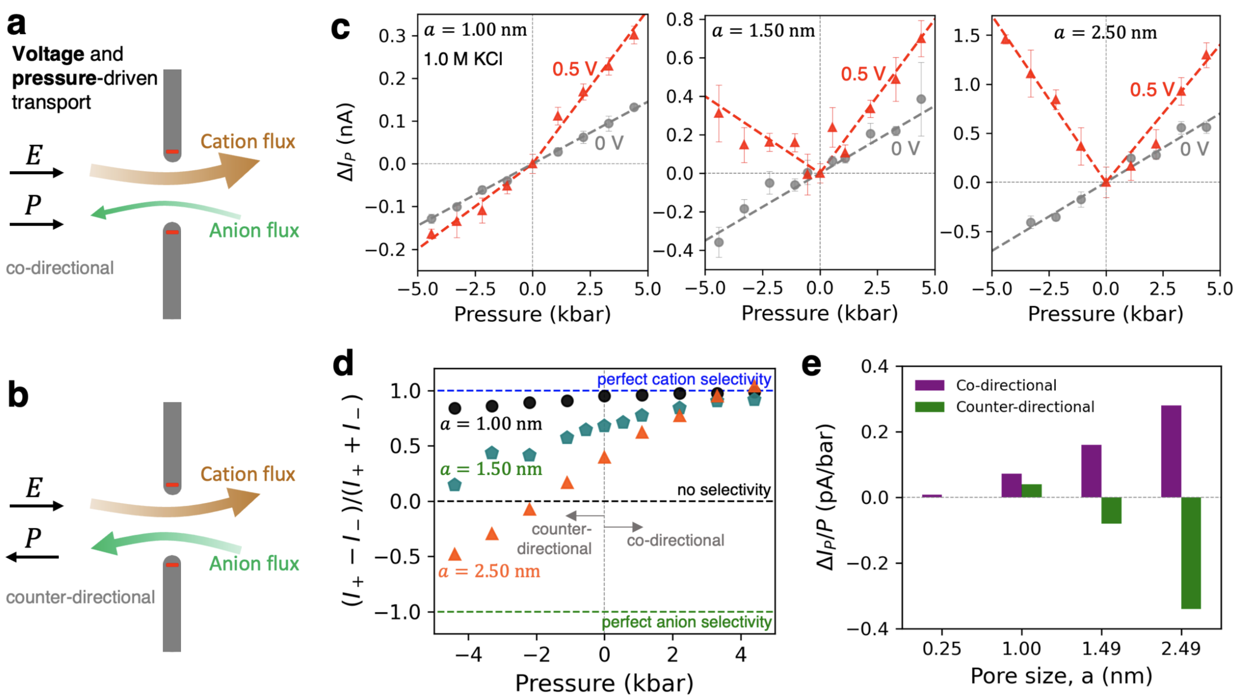

FIG. S2: **Voltage- and pressure-driven ion transport for various pore sizes** Sketch of pressure-driven ion transport: (a) co-directional application of voltage and pressure gradients, and (b) counter-directional application of the same. (c) Ion current-pressure curves with and without the application of voltage in a 1 M KCl solution. Three different pore sizes are considered: 1.0 nm, 1.5 nm, and 2.5 nm, respectively. (d) Selectivity between cations and anions as a function of pressure for three different pore sizes. (e) Voltage-induced enhancement of ion current normalized by pressure for different pore sizes.

is once again several orders of magnitude of the "baseline" current through the same pores caused by the electrostatic bias alone. The value of ΔI_P in the typical experimental pressure range ($|P| \lesssim 10$ bar) is in the $\lesssim 5$ pA range, which is two to three orders of magnitude smaller than the voltage-driven current at 0.5V. Although the same effect may be more significant for considerably larger pores that carry nonzero charge, it is vanishing for dipolar pore edges within experimentally relevant pressure levels. We note that although hydrodynamic slip can boost the coupling in the case of graphene [2], it should not be significant for hBN [8].

ION FLUX AND CURRENT CALCULATIONS

The ion currents reported in this work were calculated based on cumulative fluxes, as shown in Fig. S3, along with the flux calculation scheme (in the inset). For every ion in the system, its Z-position was tracked. Crossing events for the regions below and above membrane were determined based on the comparison between current and previous position of each ion. The flux presented here, as well as all fluxes underlying the ion currents reported in this work, were calculated using $h = 0.8$ nm. Note that the value of h only affects the amount of flux noise and in principle these calculations can be performed with $h = 0$, in which case the flux counters are simply updated by ± 1 for each crossing of the membrane. As presented, the current is the slope of the linear fit to the raw flux data. The single-pore current is then obtained from rescaling the result by the number of pores in the membrane.

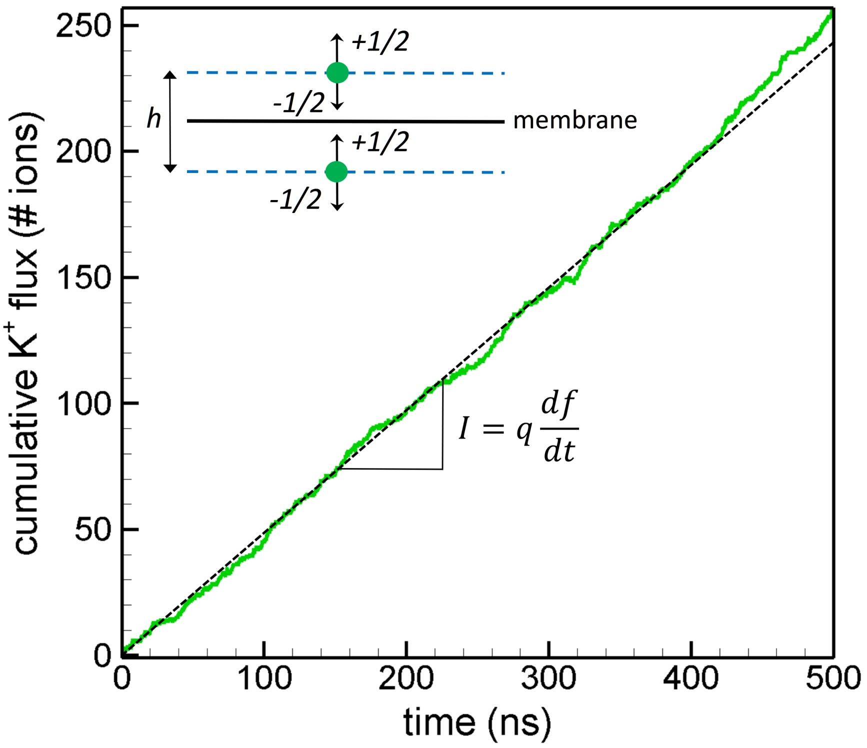

FIG. S3: **Ion flux calculations** Cumulative K^+ flux through an hBN membrane featuring a 3×3 array of B_3N pores, simulated under an external electrostatic field of $E_z = 0.01667$ V/nm, which corresponds to an estimated potential difference of $V = E_z \times L_z \approx 0.1$ V ($L_z \approx 6$ nm is the size of the simulation box size along Z) for 500 ns. The inset at the top shows the simple flux updating scheme employed in this work. Membrane location is typically close to $L_z/2$.

PORE LENGTH ESTIMATION

The effective pore length $L \approx 0.5$ nm used to obtain the analytical curves in Fig. 2c of the main text was estimated as the approximate thickness of an MD-simulated water-inaccessible region shown in Fig. S4.

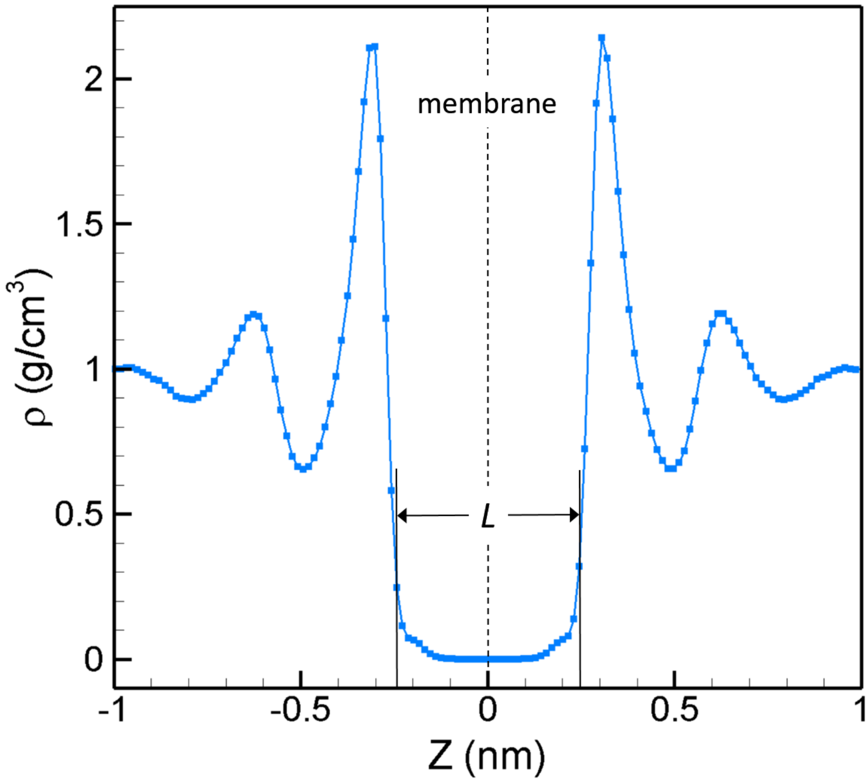

FIG. S4: **Pore length estimate** Water density distribution along the Z-direction. Each data point is the average over a planar XY-bin at the corresponding Z-location. As shown, the membrane is located at $Z = 0$.

CURRENT-STRAIN RESPONSE FOR A LARGE PORE

Mechanosensitive response was investigated for aqueous KCl and NaCl transport through a 2.5-nm-wide pore to demonstrate similar values of μ for wider pores regardless of the cation type. As shown in Fig. S5, we report $\mu_{KCl} = 2.68$ and $\mu_{NaCl} = 2.51$. Per the definition of mechanosensitivity given in the main text, here it was calculated as $\mu = \frac{k}{\alpha I_0}$, where k is the slope of the corresponding linear fit (solid line), I_0 is the current at $\epsilon = 0$, and $\alpha \approx 2$ is as defined in the discussion accompanying Eq. 5 in the main text.

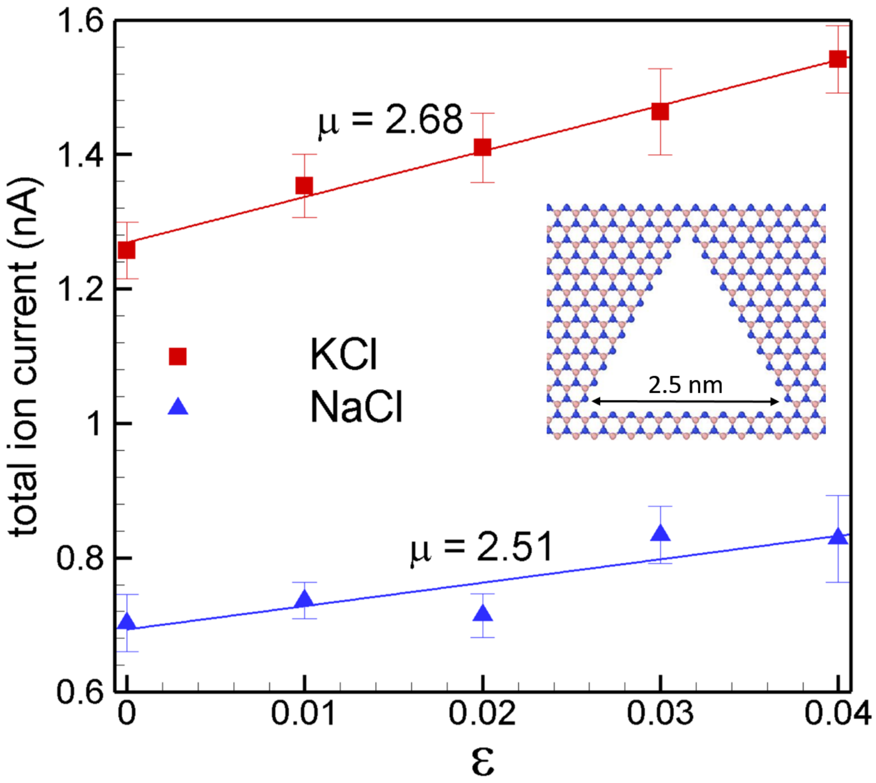

FIG. S5: **Mechanosensitive response for a 2.5-nm-wide pore** Total ionic current through a 2.5-nm-wide pore as a function of isotropic XY-strain. Simulations were set up similarly to those described in the main text; 0.5 M KCl and NaCl salts were used. The bias voltage was 0.18 V ($E_Z = 0.03$ V/nm). The solid lines are linear fits to simulated datasets.

[1] S. J. Davis, M. Macha, A. Chernev, D. M. Huang, A. Radenovic, and S. Marion, Nano Letters **20**, 8089 (2020).

[2] X. Jiang, C. Zhao, Y. Noh, Y. Xu, Y. Chen, F. Chen, L. Ma, W. Ren, N. R. Aluru, and J. Feng, Science Advances **8**, eabj2510 (2022).

[3] F. H. van der Heyden, D. Stein, and C. Dekker, Physical Review Letters **95**, 116104 (2005).

[4] Y. S. Choi and S. J. Kim, Journal of Colloid and Interface Science **333**, 672 (2009).

[5] D. Cohen-Tanugi and J. C. Grossman, Nano Letters **12**, 3602 (2012).

[6] M. Heiranian, A. B. Farimani, and N. R. Aluru, Nature Communications **6**, 8616 (2015).

[7] Y. Noh and N. Aluru, Nano Letters **22**, 419 (2021).

[8] A. R. Poggioli and D. T. Limmer, The Journal of Physical Chemistry Letters **12**, 9060 (2021).
